# Supplementary material for: The Questionable Quality Profile of Food Supplements: The Case of Red Yeast Rice Marketed Products
Source: Foods. 2023 May 25;12(11):2142. doi: 10.3390/foods12112142 (PMC10252911; doi:10.3390/foods12112142)
Supplement: Supplementary file 1 [file foods-12-02142-s001.zip › foods-2416139-supplementary.pdf]

foods

Supplementary materials to:

# The Questionable Quality Profile of Food Supplements: The Case of Red Yeast Rice Marketed Products

**Antonella Vitiello [1, †], Luana Izzo [2, †], Luigi Castaldo [2], Ivana d'Angelo [3], Francesca Ungaro [1], Agnese Miro [1,*], Alberto Ritieni [2] and Fabiana Quaglia [1]**

[1] Drug delivery Laboratory, Department of Pharmacy, University of Naples Federico II, Via Domenico Montesano 49, 80131, Naples, Italy; antonella.vitiello3@unina.it (A.V.); francesca.ungaro@unina.it (F.U.); fabiana.quaglia@unina.it (F.Q.)
[2] FoodLab Laboratory, Department of Pharmacy, University of Naples Federico II, Via Domenico Montesano 49, 80131 Naples, Italy; luana.izzo@unina.it (L.I.); luigi.castaldo2@unina.it (L.C.); alberto.ritieni@unina.it (A.R.)
[3] Department of Environmental, Biological and Pharmaceutical Sciences and Technologies, University of Campania "Luigi Vanvitelli", Via Antonio Vivaldi 43, 81100 Caserta, Italy; ivana.d'angelo@unicampania.it (I.d.A)
* Correspondence: agnese.miro@unina.it (A.M.); Tel.: +39 081678615
† These authors contributed equally to this work.

foods MDPI

**Table S1.** Experimental condition and specification of mass uniformity assay prescribed by Ph. Eur. 11. Ed.

| Dosage Form | Average Mass | Percent deviation |
|---|---|---|
| Tablets (uncoated and film-coated) | Not more than 80 mg | 10 |
| | More than 80 mg and less than 250 mg | 7.5 |
| | 250 mg or more | 5 |
| Capsules | Less than 300 mg | 10 |
| | 300 mg or more | 7.5 |

**Table S2**. Experimental conditions and specification for pharmacopeial disintegration test.

| | European Pharmacopoeia 11 | |
|---|---|---|
| **Dosage form** | **Immersion liquid/disc** | **Maximum time of Disintegration** |
| Uncoated tablets | Purified water (35-39°C) with disc | 15 min |
| Coated tablets | Purified water (35-39°C) with disc [a] | 30 min (film-coated) 60 min (other coatings) |
| Gastro-resistant tablets | HCl 0.1 M (35-39°C) without disc and then | Intact after not less than 1h |
| | Phosphate buffer solution pH 6.8 (35-39°C) with disc [b] | 60 min |
| Hard capsules | Purified water or HCl 0.1 M [c] | 30 min |
| Soft capsules | Purified water or HCl 0.1 M with disc [d] | 30 min |

[a] If any of the tablets has not disintegrated, the test is repeat on a further 6 tablets, replacing water R with 0.1 M hydrochloric acid.
[b] If the tablets are not compliant to the test, due to disc adherence, the test should be repeated without discs.
[c] The use of discs is required if the capsules float in the immersion liquid.
[d] If the sof capsule adheres to the disc, it can be omitted.

**Table S3**. Stock solutions composition.

| Salt solution | Stock concentration (mol/L) | SSF (pH 7) mL of Stock added to prepare 0.4 L (mL) | SSF (pH 7) Final salt concentration in sample (mmol/L) | SGF (pH 3) mL of Stock added to prepare 0.4 L (mL) | SGF (pH 3) Final salt conc. in sample (mmol/L) | SIF (pH 7) mL of Stock added to prepare 0.4 L (mL) | SIF (pH 7) Final salt conc. in sample (mmol/L) |
|---|---|---|---|---|---|---|---|
| KCl | 0.5 | 15.1 | 15.1 | 6.9 | 6.9 | 6.8 | 6.8 |
| $KH_2PO_4$ | 0.5 | 3.7 | 1.35 | 0.9 | 0.9 | 0.8 | 0.8 |
| $NaHCO_3$ | 1 | 6.8 | 13.68 | 12.5 | 25 | 42.5 | 85 |
| NaCl | 2 | - | - | 11.8 | 47.2 | 9.6 | 38.4 |
| $MgCl_2(H_2O)_6$ | 0.15 | 0.5 | 0.15 | 0.4 | 0.12 | 1.1 | 0.33 |
| $NH_4(CO_3)_2$ | 0.5 | 0.06 | 0.06 | 0.5 | 0.5 | - | - |
| $CaCl_2(H_2O)_2$ | 0.3 | - | 1.5 | - | 0.15 | - | 0.6 |

Abbreviations: SSF: simulated salivary fluid, SGF: simulated gastric fluid, and SIF: simulated intestinal fluid.

**Table S4.** Information relevant for the study reported on the label of FS.

| Sample | Dosage form category | Single unit weight [a] (mg) | Number of AIs | AIs (Monacolin K) (mg) | Inactive ingredients (mg) |
|---|---|---|---|---|---|
| #1 | Film-coated tablet | 400 | 7 | 290 (-) [d] | 110 |
| #2 | Film-coated tablet | 1000 | 2 | 539 (1.45) [b] | 471 |
| #3 | Film-coated tablet | 1100 | 3 | 615 (10) [b] | 385 |
| #4 | Film-coated tablet | 1340 | 4 | 1004 (5) [b] | 336 |
| #5 | Film-coated tablet | 983 | 6 | 780 (-) [c] | 203 |
| #6 | Uncoated tablet | 1000 | 1 | 667 (-) [d] | 333 |
| #7 | Uncoated tablet | 550 | 5 | 320 (10) [b] | 230 |
| #8 | Uncoated tablet | Not reported | 8 | 739 (10) [b] | - |
| #9 | Uncoated tablet | 330 | 1 | 200 (10) [b] | 130 |
| #10 | Hard shell capsule | 450 | 2 | 238.7 (10) [b] | 211 |
| #11 | Hard shell capsule | 500 | 2 | 280 (10) [b] | 220 |
| #12 | Hard shell capsule | 450 | 6 | 213 (2.2) [b] | 237 |
| #13 | Softgels | 1600 | 8 | 1063 (10) [b] | 537 |
| #14 | Softgels | 1777 | 4 | 658 (5) [b] | 1120 |

[a] Calculated from the ratio between the total weight declared on the label and the number of dosage form units.
[b] Reported on the label as monacolin K.
[c] Reported on the label as monacolins.
[d] Not specified.
AI= Active ingredient.
Note. Following COMMISSION REGULATION (EU) 2022/860, the use of monacolins from red yeast rice is allowed at levels of less than 3 mg per portion of the product recommended for daily consumption.

**Table S5.** Inactive ingredients listed on the RYR FS label.

| | | Film-coated tablets | | | | | Uncoated tablets | | | |
|---|---|---|---|---|---|---|---|---|---|---|
| | | **#1** | **#2** | **#3** | **#4** | **#5** | **#6** | **#7** | **#8 [a]** | **#9** |
| Tablet/tablet core | Microcrystalline cellulose | X | X | X [b] | X [b] | X | X [b] | X | - | X [b] |
| | Starch | | | | | | X | | - | |
| | Maltodextrines from Maize | | X | | | | | | - | |
| | Calcium Carbonate | | | | | | X | | - | |
| | Cross-linked sodium carboxymethylcellulose | | X | X | X | X | | | - | |
| | Silicon dioxide | | X | X | X | X | X | X | - | X |
| | Magnesium salts of fatty acids | X [c] | X | X | X | X | X | X | - | X |
| | Carminic Acid | | | | X | | | | - | |
| | Titanium dioxide (E. 171) [d] | | X | X | | | | | - | |
| | Iron oxide red | | | X | | | | | - | |
| | Iron oxide black | | | | | | | | - | |
| | Iron oxides and iron hydroxides | | X | | X | | | | - | |
| Coating | Hydroxypropyl methyl cellulose | X | X | X | | | | | - | |
| | Hydroxypropylcellulose | | | | | X | | | - | |
| | Polyvinyl alcohol | | | X | | | | | - | |
| | Polyvinylpyrrolidone | | | | | X | | | - | |
| | Microcrystalline cellulose | X | X | X | X | X | | | | |
| | Talc | | X | | X | X | | | - | |
| | Polyethylene glycol | | X | | X | | | | - | |
| | Glycerol | | | X | | | | | - | |
| | Stearic acid | X | | | | | | | - | |
| | Polysorbate 80 | | | | X | | | | - | |
| | Niacin (nicotinamide/vitamin B3) | | | | X | | | | - | |
| | Shellac | | | | | X | | | - | |

[a] The label does not report the ingredient list.
[b] Reported as "cellulose" in the ingredient list.
[c] Reported as "vegetable magnesium stearate" in the ingredient list.
[d] Banned in food since 7th August 2022.

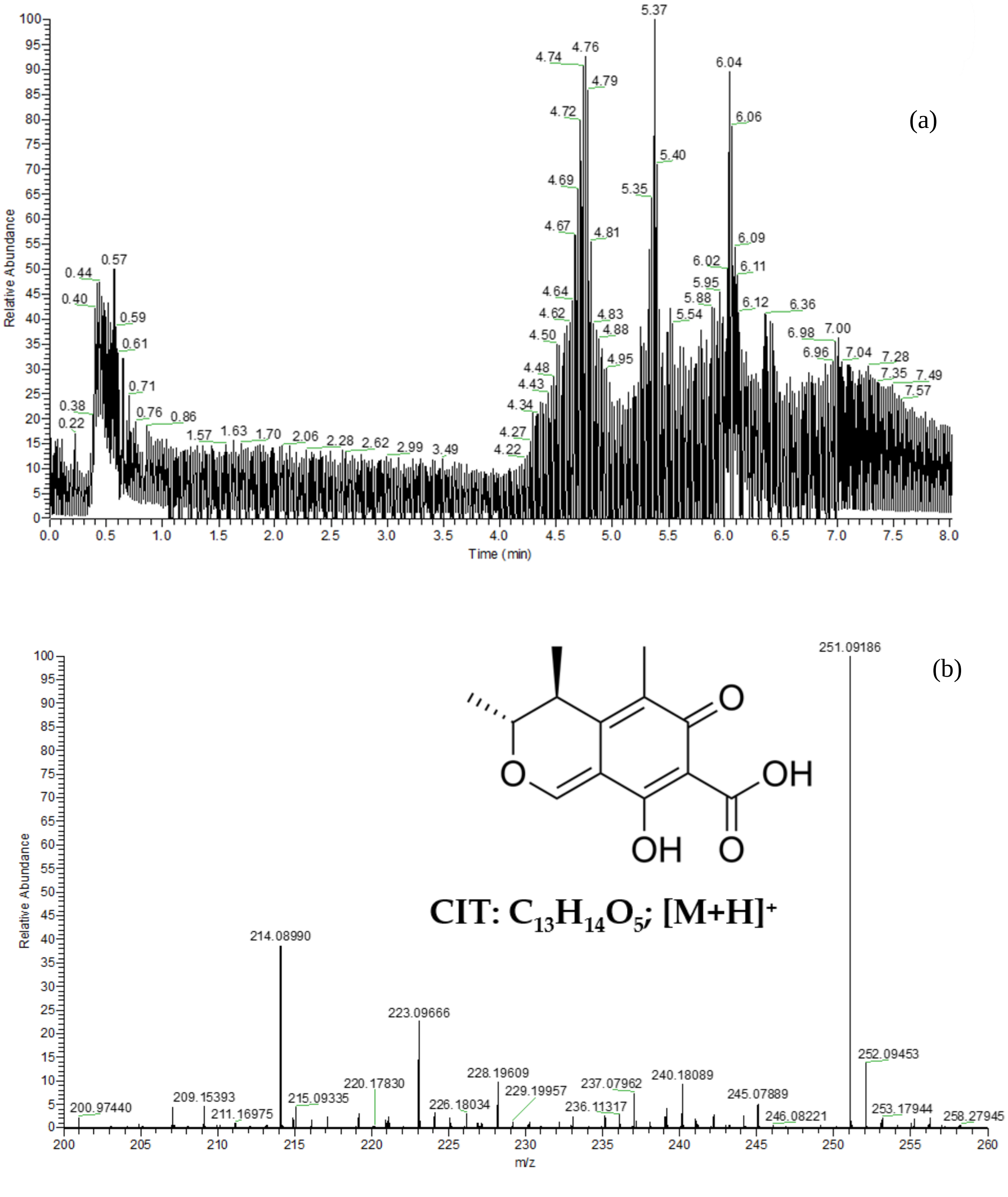


**Figure S1.** Extracted ion chromatogram (a) and mass spectra (b) of citrinin
